# Supplementary material for: Multidisciplinary Management of Suspected Lyme Borreliosis: Clinical Features of 569 Patients, and Factors Associated with Recovery at 3 and 12 Months, a Prospective Cohort Study
Source: Microorganisms. 2022 Mar 12;10(3):607. doi: 10.3390/microorganisms10030607 (PMC8955660; doi:10.3390/microorganisms10030607)
Supplement: Supplementary file 1 [file microorganisms-10-00607-s001.zip › microorganisms-1520731-supplementary.pdf]

Supplementary file S1. Sensibility analysis: Univariate and multivariate analyses of the associated factors of rapid recovery versus partial improvement at 3 months after care at the TBD-RC – Paris and Northern region

			Univariate analysis		Multivariate analysis	
**Risk factor**	N (n=389)	n(%) cured patients at 3 months	OR [95% CI]	P value	OR [95% CI]	P value
**Age**				0.20		0.26
<35 years-old	102	39 (38.2)	1		1	
35-48 years-old	97	25 (25.8)	0.56 [0.31-1.03]		0.56 [0.29-1.06]	
48-61 years-old	92	29 (31.5)	0.74 [0.41-1.35]		0.72 [0.38-1.38]	
>61 years-old	98	37 (37.8)	0.98 [0.55-1.73]		0.95 [0.51-1.76]	
**Sex**				0.07		0.98
Male	146	57 (39.0)	1.49 [0.97-2.30]		1.01 [0.63-1.60]	
Female	243	73 (30.0)	1		1	
**History of tick-bite**				0.51	-	-
Yes	273	94 (34.4)	1.17 [0.73-1.86]			
No	116	36 (31.0)	1			
**History of erythema migrans**				0.12	-	-
Yes	115	45 (39.1)	1.43 [0.91-2.25]			
No	274	85 (31.0)	1			
**Serology**				0.014	-	-
Positive serology in ELISA and WB	132	45 (34.1)	1.30 [0.80-2.11]			
Positive serology in ELISA only	54	19 (35.2)	1.36 [0.71-2.60]			
Negative serology in ELISA	179	51 (28.5)	1			
Patient with no serology (erythema migrans)	24	15 (62.5)	4.18 [1.72-10.16]			

**Delay 1st symptoms - 1st consultation at the TBD-RC**				0.001		0.001
0-155 days (0.0 – 0.4 year)	108	53 (49.1)	1		1	
155-512 days (0.4 – 1.4 years)	107	41 (38.3)	0.64 [0.37-1.11]		0.88 [0.49-1.56]	
512-1393 days (1.4 – 3.8 years)	91	24 (26.4)	0.37 [0.20-0.68]		0.51 [0.27-0.99]	
>1393 days (> 3.8 years)	82	12 (14.6)	0.18 [0.09-0.37]		0.22 [0.10-0.47]	
**Delay 1st consultation at the TBD-RC - final diagnosis**				0.001		0.001
0 day	163	80 (49.1)	1		1	
1-15 days	25	10 (40.0)	0.69 [0.29-1.63]		0.62 [0.26-1.51]	
15-83 days	105	23 (21.9)	0.29 [0.17-0.51]		0.28 [0.15-0.50]	
>83 days	96	17 (17.7)	0.22 [0.12-0.41]		0.27 [0.14-0.52]	
**Final diagnosis**				0.012		0.22
Proven LB	66	29 (43.9)	1.47 [0.85-2.56]		0.93 [0.49-1.77]	
Possible LB	32	9 (28.1)	0.74 [0.33-1.66]		1.13 [0.46-2.75]	
PTLDS or sequelae	49	8 (16.3)	0.37 [0.16-0.82]		0.40 [0.17-0.96]	
Differential diagnosis	242	84 (34.7)	1		1	
**Number of diagnosis per patient**				0.001		0.004
1 diagnosis	210	93 (44.3)	1		1	
2 diagnoses	106	24 (22.6)	0.37 [0.22-0.63]		0.43 [0.25-0.75]	
>3 diagnoses	73	13 (17.8)	0.27 [0.14-0.53]		0.46 [0.23-0.93]	
**History of antibiotics prescribed before the TBD-RC**				0.23	-	-
Yes	261	82 (31.4)	0.76 [0.49-1.19]			
No	128	48 (37.5)	1			
**History of non-recommended antibiotics before the TBD-RC**				0.001		0.023

Yes	64	10 (15.6)	0.32 [0.15-0.64]		0.41 [0.19-0.88]	
No	325	120 (36.9)	1		1	
**First line of antibiotics prescribed at the TBD-RC**				0.07	-	-
Yes	117	47 (40.2)	1.53 [0.97-2.40]			
No	272	83 (30.5)	1			
**Second line of antibiotics at the TBD-RC**				0.69	-	-
Yes	14	4 (28.6)	0.79 [0.24-2.57]			
No	375	126 (33.6)	1			

TBD-RC = Tick-Borne Diseases Reference Center ; PTLDS = Post-Treatment Lyme Disease Syndrome
